# Programmable HMO biosynthesis enabled by human cell-based glycoengineering

**DOI:** 10.1093/glycob/cwag066

**Published:** 2026-08-19

**Authors:** Stijn Kruf, Roy J B M Delahaije, Morihisa Fujita, Christian Büll

**Affiliations:** Department of Biomolecular Chemistry, Institute for Molecules and Materials, Radboud University, Heyendaalseweg 135, 6525 AJ, Nijmegen, the Netherlands; FrieslandCampina, Stationsplein 4, 3818 LE, Amersfoort, the Netherlands; Institute for Glyco-core Research (iGCORE), Gifu University, 1-1 Yanagido, Gifu 501-1193, Japan; Department of Biomolecular Chemistry, Institute for Molecules and Materials, Radboud University, Heyendaalseweg 135, 6525 AJ, Nijmegen, the Netherlands

**Keywords:** a-lactalbumin, glycoengineering, glycosyltransferase, human milk oligosaccharides, lactose

## Abstract

The lactating mammary gland is uniquely adapted to synthesize unconjugated free glycans known as human milk oligosaccharides (HMOs). HMO biosynthesis is initiated by the formation of lactose, the common precursor from which structurally diverse neutral and acidic oligosaccharides are generated through the sequential action of Golgi-resident glycosyltransferases. Although microbial fermentation and chemical synthesis have enabled production of selected HMOs, human cell-based platforms remain largely unexplored despite providing the native glycosylation machinery required for HMO assembly. Consequently, the contributions of individual glycosyltransferases to HMO biosynthesis have remained difficult to define. Here, we describe the construction of HMO biosynthesis in human embryonic kidney (HEK293) cells through constitution of the lactose synthase complex and systematic engineering of downstream glycosyltransferases. This modular approach enables programmable production of structurally defined HMOs, including both simple sialylated oligosaccharides and more complex type-I and type-II structures, while providing a tractable platform for assigning glycosyltransferase functions within the HMO biosynthetic pathway. Together, this establishes mammalian glycoengineering as a versatile system for investigating HMO biosynthesis and for the tailored production of biologically relevant HMOs.

## Introduction

Human milk oligosaccharides (HMOs) are a group of unconjugated carbohydrates found in human breast milk and represent the third most abundant group after lactose and fat (Bode 2012). All HMOs are composed of 3 to 5 of the main structural building blocks glucose (Glc), galactose (Gal), N-acetylglucosamine (GlcNac), fucose (Fuc) and sialic acid (Sia), with *N*-acetylneuraminic acid (Neu5Ac) as the predominant form of Sia (Mainardi et al. 2023). HMOs play a central role in early-life development by shaping the intestinal microbiota, preventing pathogen adhesion, modulating immune responses, and supporting epithelial barrier function (Triantis et al. 2018; Sprenger et al. 2022; Dinleyici et al. 2023). Increasing evidence also links specific HMOs to neurodevelopment and protection against diseases such as necrotizing enterocolitis (Sprenger et al. 2022). These biological activities have stimulated considerable interest in incorporating HMOs into infant nutrition and developing HMO-based therapeutics.

To address the growing demand for defined HMOs, chemical, chemoenzymatic, and microbial production platforms have been developed (Xiao et al. 2016; Prudden et al. 2017; Agoston et al. 2019; Zhu et al. 2022; Urashima et al. 2025). Among these, microbial fermentation has become the predominant manufacturing strategy for several commercially available HMOs, including 2′-fucosyllactose (2'-FL), 3′-sialyllactose (3'-SL), and lacto-*N*-neotetraose (LNnT) (Pressley et al. 2024). Nevertheless, construction of the complete HMO biosynthetic network in microbial hosts remains challenging because bacteria lack the mammalian Golgi glycosylation machinery, requiring extensive engineering of nucleotide sugar metabolism, glycosyltransferases, and transport systems (Zeuner et al. 2019; Gan et al. 2023). Consequently, production of larger and more complex HMOs remains technically demanding.

Human cells provide an attractive alternative because they naturally express many of the metabolic genes, glycosyltransferases, nucleotide sugar transporters, and secretory pathways required for HMO biosynthesis. Yet, human cell lines lack the capacity to generate free oligosaccharides. The major bottleneck is a lack of α-lactalbumin (LALBA) expression that is restricted to the lactating mammary gland (Turkington et al. 1968; Hannan et al. 2023). LALBA heterodimerizes with β1–4-galactosyltransferase 1 (B4GALT1) into the lactose synthase complex which catalyzes the formation of lactose from glucose and UDP-galactose and thereby initiates HMO biosynthesis (Brew et al. 1968; Ebner 1966; Klee 1972). In addition, the specific contributions of several glycosyltransferases to HMO assembly remain incompletely understood because of functional redundancy within enzyme families and the absence of tractable mammalian model systems for pathway construction (Kellman et al. 2022; McDonald et al. 2022; Mohamed et al. 2024).

We have previously developed a programmable mammalian platform for HMO biosynthesis by constituting lactose synthesis and systematically engineering downstream glycosyltransferase pathways (Kruf et al. 2026; Noda et al. 2026). Introduction of the lactose synthase complex establishes de novo HMO production in cultured mammalian cells and targeted manipulation of glycosyltransferase expression enables selective synthesis of structurally defined HMOs ranging from simple sialylated oligosaccharides to complex type-I and type-II HMOs. Furthermore, the platform supports metabolic glycan engineering and produces biologically active HMOs with prebiotic properties, illustrating its utility for both mechanistic studies of HMO biosynthesis and the production of next-generation HMOs. The findings summarized in Fig. 1 are adapted from our previous study (Noda et al. 2026), and the glycoengineered cell lines and experiments presented in Fig. 2 are original data obtained with the methods described by Kruf et al. (Kruf et al. 2026). This Technical Note integrates these findings to illustrate the potential of programmable HMO biosynthesis in human cells.

**Figure 1:**
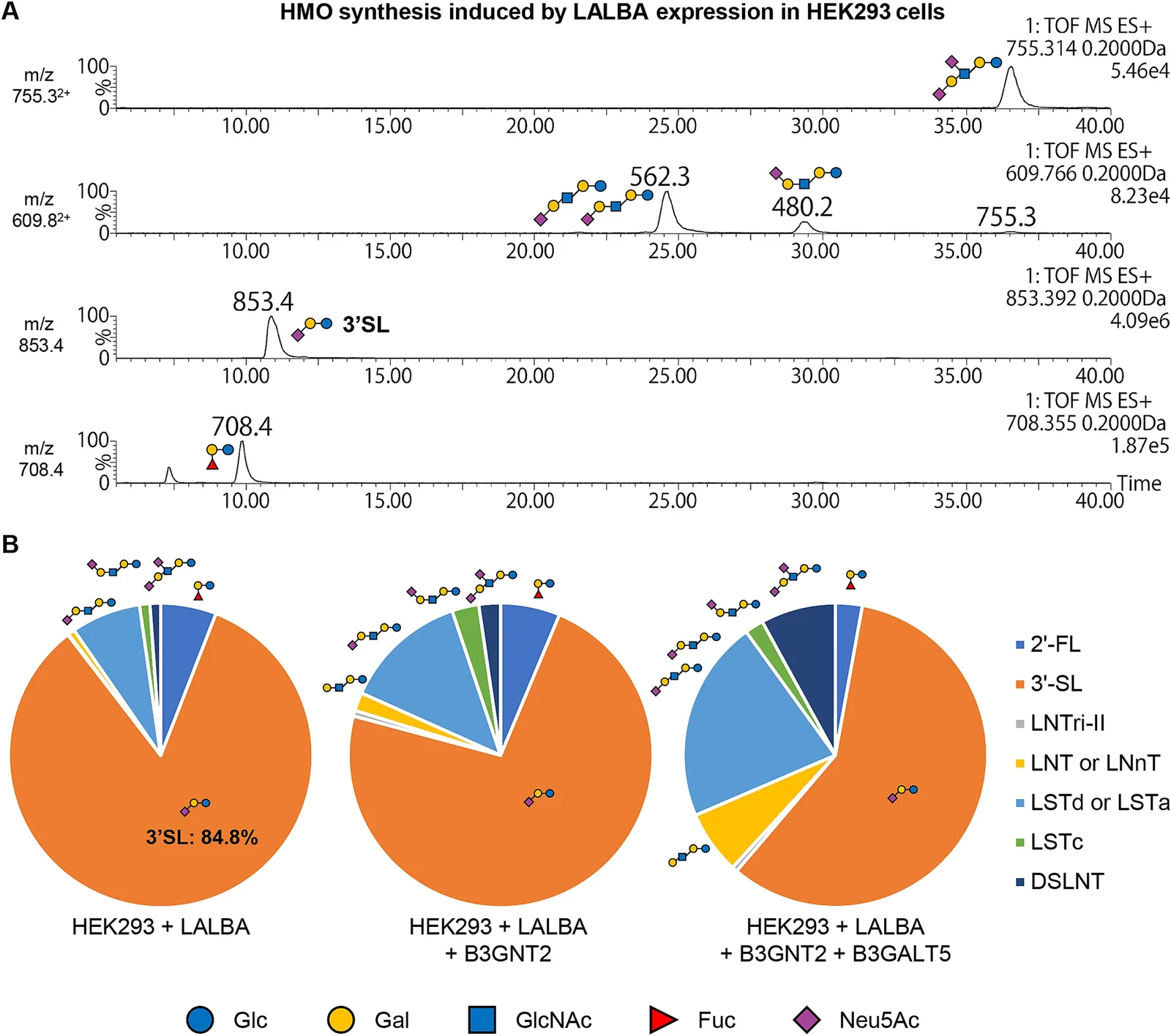
Human milk oligosaccharide (HMO) synthesis induced by α-lactalbumin (LALBA) expression in HEK293 cells. **A)** HMOs were extracted from the culture supernatants of HEK293 cells stably expressing LALBA, labeled with procainamide (ProA), purified, and analyzed by LC-ESI-MS/MS. representative extracted ion chromatograms are shown for the indicated precursor ions (m/z 755.3^2+^, 609.8^2+^, 853.4^+^, and 708.4^+^). The HMO assignments were confirmed by accurate mass, MS/MS fragmentation, and glycosidase digestions (Noda et al. 2026). **B)** Relative abundance of HMOs produced by cells expressing LALBA (*n* = 10), LALBA plus B3GNT2 (*n* = 6), or LALBA plus B3GNT2 and B3GALT5 (*n* = 6). Semi-quantitative analysis was based on the peak areas of ProA-labeled glycans detected by LC–MS. monosaccharide symbols follow the symbol nomenclature for glycans (SNFG): Glucose (Glc; blue circle), galactose (gal; yellow circle), *N*-acetylglucosamine (GlcNAc; blue square), fucose (Fuc; red triangle), and *N*-acetylneuraminic acid (Neu5Ac; purple diamond). The images were adapted from Noda et al., *metabolic engineering* 97, 102,494, 2026, under the creative commons CC BY 4.0 license (Noda et al. 2026).

**Figure 2:**
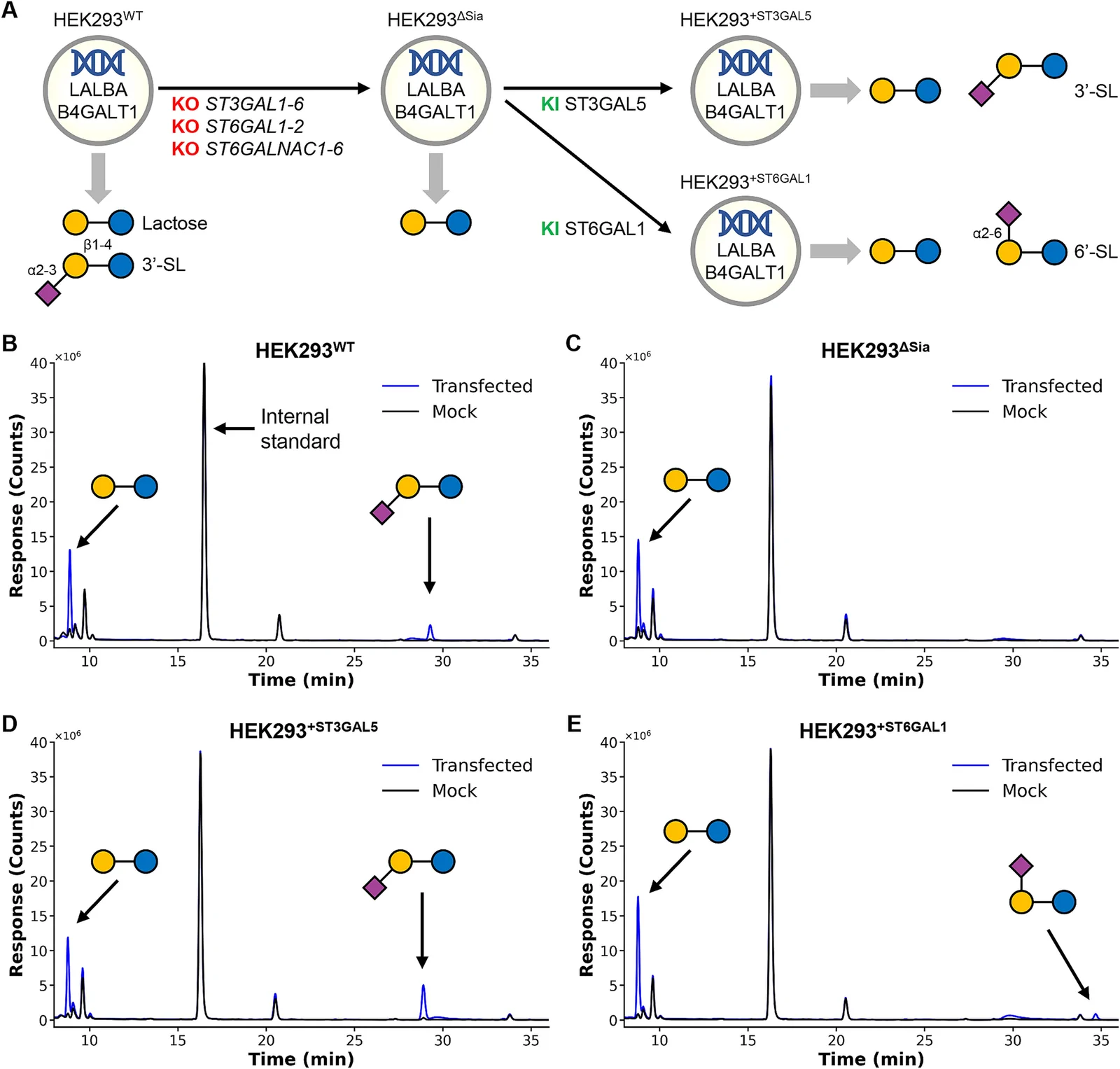
Sialyllactose expression in glycoengineered HEK293 cells. **A)** Schematic overview of HEK293 cell lines with stable genetic knock-out (KO) and knock-in (KI) of sialyltransferases and the type of HMO produced upon co-transfection with α-lactalbumin (LALBA) and β-1 4-galactosyltransferase 1 (B4GALT1). **B**-**E**) Representative chromatograms show human milk oligosaccharide (HMO) production in different HEK293 cell lines co-transfected with LALBA and B4GALT1 or mock-transfected controls. Supernatants were collected 72 h post-transfection and subjected to 2-aminobenzamide labeling (2-AB) followed by ultra-performance liquid chromatography analysis with fluorescent detection (UPLC-FD). Arrows at peak areas indicate lactose (retention time ~ 8.8 min), 3'-sialyllactose (3’-SL,retention time ~ 29.3 min), and 6'-sialyllactose (6’-SL,retention time ~ 34.7 min). Chromatograms are shown for wild type HEK293 cells (HEK293^WT^) **(B)**, HEK293^ΔSia^ cells that lack endogenous sialylation capacity **(C)**, HEK293^+ST3GAL5^ cells that express ST3GAL5 as only sialyltransferase **(D),** and HEK293^+ST6GAL1^ cells that express ST6GAL1 as only sialyltransferase (**E**). Glycan symbols are drawn according to the symbol nomenclature for glycans format (Varki et al. 2015). The data are original.

## Results

### Expression of **α**-lactalbumin enables de novo biosynthesis of multiple HMOs in HEK293 cells

Most mammalian cell lines lack endogenous capacity for production of HMOs due to the lack of LALBA expression that forms lactose synthase together with B4GALT1 (Brew et al. 1968; Ebner 1966; Klee 1972). Human embryonic kidney cells (HEK293) that are widely used for recombinant glycoprotein production display B4GALT1 that adds galactose to N-glycans (Narimatsu et al. 2019), but they lack LALBA expression which is needed to shift substrate recognition to free glucose (Ramakrishnan et al. 2001). To determine whether expression of LALBA complex is sufficient to initiate HMO biosynthesis, we transfected LALBA into wild type (WT) HEK293^WT^ cells. Free glycans in conditioned culture supernatant were subjected to 2-aminobenzamide labeling or procainamide labeling and we were able to identify several HMOs with ultra-performance liquid chromatography analysis with fluorescent detection (UPLC-FD) and tandem mass spectrometry combined with liquid chromatography (LC–MS/MS) analysis respectively. While HMO detection was absent in the conditioned medium of mock-transfected HEK293 cells, LALBA expression induced HMOs. Extracted ion chromatograms identified products with m/z values corresponding to 2′-FL, 3′-SL, sialyllacto-*N*-tetraose (LST), and disialyllacto-*N*-tetraose (DSLNT) (Fig. 1A). Semi-quantitative analysis showed that 3′-SL was the predominant product, accounting for approximately 85% of total HMO production, whereas 2′-FL, LSTd/LSTa, LSTc, DSLNT, and neutral tetraose structures lacto-N-tretraose (LNT) and LNnT were present at substantially lower abundance (Fig. 1B). The data presented in Fig. 1 are reproduced from (Noda et al. 2026) and demonstrate that introduction of LALBA is sufficient to activate endogenous HMO biosynthesis and that endogenous glycosyltransferases in HEK293 cells preferentially direct lactose toward α2–3-sialylation.

### Steering HMO biosynthesis through genetic glycoengineering

To extend HMO biosynthesis and to steer biosynthesis from 3′-SL towards more complex HMOs, we expressed additional glycosyltransferases in LALBA-expressing HEK293 cells. Overexpression of β-1,3-N-acetylglucosaminyltransferase 2 (B3GNT2), which catalyzes the formation of the Lacto-N-triose II (LNTri-II) intermediate, substantially increased the synthesis of extended HMOs, including LNT/LNnT, LSTa/LSTd, LSTc, and DSLNT. In particular, LSTa/LSTd and DSLNT increased approximately by 1.8 ± 0.1-fold and 2.1 ± 0.1-fold (mean ± SE, *n* = 6), respectively, indicating that B3GNT2 efficiently redirects metabolic flux toward elongated HMO structures (Fig. 1B). Co-expression of β-1,3-galactosyltransferase (B3GALT5) with LALBA and B3GNT2 markedly remodeled the HMO profile. 3′-SL and 2′-FL levels were reduced and we found increases in LNT and for the sialylated type-I structures LSTa and an almost 7.9 ± 1.6-fold (mean ± SE, *n* = 6) increase for DSLNT, compared to HEK293 cells expressing LALBA alone. Data are reproduced from (Noda et al. 2026).

Extended stable genetic glycoengineering to delete or express defined sets of glycosyltransferases can potentially be applied to gain more precise control of the HMO output of a cell lines. We have co-expressed LALBA and B4GALT1 in HEK293 cells with stable knock-out (KO) and knock-in (KI) of sialyltransferase family members (Fig. 2A). HEK293^WT^ cells secreted mainly lactose and 3′-SL and combined KO of the ST3GAL, ST6GAL, and ST6GALNAC families yielded cells devoid of sialylation capacity (HEK293^ΔSia^), impairing 3′-SL biosynthesis (Fig. 2B, C). LALBA/B4GALT1 co-transfection in HEK293^ΔSia^ cell with KI of ST3GAL5 yielded efficient conversion of lactose to 3’-SL (Fig. 2D). To steer HMO biosynthesis towards 6′-SL production, we employed HEK293^ΔSia^ cells with KI of ST6GAL1 (Fig. 2E). The data presented in Fig. 2 are original and were obtained using our previously described approach (Kruf et al. 2026). Together, these results demonstrate that HMO biosynthesis can be programmed in a modular manner by combinatorial introduction or deletion of individual or multiple key glycosyltransferases.

## Discussion

While the production of recombinant glycoproteins with defined glycans by HEK293 cells is advancing fast due to genetic glycoengineering (Narimatsu et al. 2021; Huang et al. 2024; Jaroentomeechai et al. 2024), the use of human cell systems to produce free oligosaccharides is lagging behind. This is at least partly related to the complexity of the HMO biosynthesis pathway and that many steps and involved enzymes are not fully elucidated (Kellman et al. 2022; McDonald et al. 2022; Nyquist et al. 2025; Slater et al. 2025).

Here, we establish a human cell-based glycoengineering platform for the programmable biosynthesis of HMOs. By establishing lactose synthesis through expression of LALBA and systematically engineering downstream glycosyltransferases, HEK293 cells can be instructed to synthesize a broad spectrum of structurally diverse HMOs. Further genetic glycoengineering inserting or deleting glycosyltransferases can direct HMO biosynthesis towards specific HMO structures such as 3′-SL and 6′-SL and more complex ones. Moreover, it enables probing individual and combined contributions of glycosyltransferases to specific steps in HMO biosynthesis. This is particularly valuable to dissect the functions of individual members of the larger glycosyltransferase families such as the galactosyltransferases and the sialyltransferases in a relevant cellular environment.

Although yields of HMO production in the HEK293 cell system can potentially be enhanced and human cells endogenously feature nucleotide sugars, transporters, and glycosyltransferases, this system is likely not competitive with current microbial HMO production platforms in terms of production capacity and costs (Yu et al. 2018; Zhang et al. 2019; Lv et al. 2024; You et al. 2024). Under the current experimental conditions, HMO production reached up to approximately 100 mg/L, which remains substantially lower than microbial production platforms that typically achieve yields in the gram-per-liter range. However, HEK293 cells allow to discover the molecular details and Golgi organization of HMO biosynthesis which potentially can guide further engineering to improve microbial systems and to enable production of more complex HMOs that are currently challenging to produce with fermentation systems.

Together, the reproduced (Fig. 1) and original data (Fig. 2) presented here position human cell-based glycoengineering as a versatile platform for both the mechanistic understanding of HMO biosynthesis and for production of complex HMOs (Kruf et al. 2026; Noda et al. 2026). Continued glycoengineering will allow the biosynthesis of increasingly complex fucosylated, sialylated, and branched HMOs which can be highly valuable for research applications to discover functional effects of HMOs.

## Materials and methods

### Cell culture and transfection

HEK293 cells (CRL-1573) were obtained from American Type Culture Collection (ATCC) and isogenic HEK293 cell lines were previously generated (Narimatsu et al. 2019; Bull et al. 2020; Bull et al. 2021). Cells were cultured in Dulbecco’s Modified Eagle Medium (DMEM) with high glucose and L-glutamine (Gibco) supplemented with 10% heat-inactivated FBS (Gibco) and 1× penicillin/streptomycin (Gibco) at 37 °C and 5% CO_2_ in a humidified incubator. Cells were passaged every 2–3 days and used until a maximum passage number of thirty after resuscitation from liquid nitrogen storage. Co-transfections with pIRES-eGFP LALBA and pIRES-eGFP B4GALT1 were performed using Lipofectamine 3000 (Thermo Fisher Scientific) as described previously (Kruf et al. 2026).

### Establishment of stable cell lines

Stable cell lines were established essentially as described previously (Noda et al. 2026). Briefly, HEK293 cells were transfected with a plasmid harboring LALBA using Lipofectamine 3000 and selected with 400 μg/mL hygromycin to obtain a cell line stably expressing LALBA. These cells were then further transfected with plasmids encoding B3GNT2 or both B3GNT2 and B3GALT5, and selected with 10 μg/mL blasticidin and, where applicable, 1 μg/mL puromycin to generate cell lines stably expressing B3GNT2 or co-expressing B3GNT2 and B3GALT5.

### Sample preparation for UPLC-FD

Supernatants from HEK293 cell cultures were collected 72 h post-transfection, centrifuged (21.000 g, 4 °C, 10 min), and transferred into clean 1.5 ml tubes. Supernatants (200 μL) were mixed with 10 μL Milli-Q water and 10 μL internal standard solution (500 mg/L laminaritriose, Megazyme). Proteins were precipitated via Carrez precipitation by addition of 10 μL Carrez solution 1 (Carl Roth) and 10 μL Carrez solution 2 (Carl Roth) followed by vortexing and centrifugation (16.000 g, 1 min, RT). After protein precipitation, 100 μL of the supernatant was transferred to a clean 1.5 mL tube and mixed with 100 μL label solution, which consisted of 34.67 mM 2-aminobenzamide (Sigma-Aldrich) and 1 M picoline borane (SigmαAldrich) in an acetic acid-DMSO mixture (3:7, v/v). Samples were incubated at 65 °C for 1 h under gentle shaking. Afterwards, the samples were cooled down to RT, centrifuged (16.000 g, 1 min, RT), and 1 mL 75% acetonitrile (LiChrosolv, VWR) in Milli-Q water was added to each sample. The samples were filtered over a 0.22 μm Millex PVDF syringe filter (Phenomenex) and 2 μL was injected on a Thermo Scientific Vanquish UPLC system equipped with a Waters Acquity BEH Premier Glycan column (130 Å, 1.7 μm, 2.1 × 150 mm) thermostated at 65 °C. Elution was performed at a flow rate of 0.5 mL/min using the following gradient of 50 mM ammonium formate pH 4.40 (Eluent A) and 100% ACN (Eluent B): 0–38 min linear gradient from 90 to 80.1% B, 38–38.5 min linear gradient from 80.1 to 20% B, 38.5–41.5 min isocratic on 20% B, 41.5–42 min linear gradient from 20 to 90% B, 42–52 min isocratic on 90% B. Elution was monitored using a Thermo Scientific Vanquish fluorescence detector with an emission and excitation wavelength of 260 and 420 nm, respectively.

### Sample preparation for LC–MS/MS

For HMO analysis using mass spectrometry, cells (4 × 10^5^) were seeded into 6-well plates, cultured for 1 day, and then switched to 2 mL of Opti-MEM I for a further 4 days. Culture supernatants were treated with glucose oxidase to remove residual glucose, and the released glycans were purified using BlotGlyco (Sumitomo Bakelite) and labeled with procainamide (Noda et al. 2026). The procainamide-labeled HMOs were analyzed on a SYNAPT XS LC–MS/MS system (Waters) equipped with an XBridge Glycan BEH Amide column (130 Å, 2.5 μm, 2.1 × 150 mm; Waters). HMOs were structurally annotated based on accurate mass, MS/MS fragmentation, and glycosidase digestion.

#### Data visualization and statistical analysis

Figures were processed using Python 3, Microsoft PowerPoint (Microsoft Corporation), and Excel (Microsoft Corporation). Chromatographic raw data files were processed using the Chromeleon Chromatography Data System (Thermo Fisher Scientific). Peaks were integrated manually via the valley-to-valley integration method, yielding the raw area under the curve value (AUC) and the retention time (RT).

## Data Availability

All data presented in the figures and tables, and supplementary information of this paper are available.
